# Bird diversity in northern Myanmar and conservation implications

**DOI:** 10.24272/j.issn.2095-8137.2017.059

**Published:** 2017-09-18

**Authors:** Ming-Xia Zhang, Myint Kyaw, Guo-Gang Li, Jiang-Bo Zhao, Xiang-Le Zeng, Kyaw Swa, Rui-Chang Quan

**Affiliations:** ^1^Southeast Asia Biodiversity Research Institute, Chinese Academy of Sciences, Yezin Nay Pyi Taw 05282, Myanmar; ^2^Center for Integrative Conservation, Xishuangbanna Tropical Botanical Garden, Chinese Academy of Sciences, Mengla Yunnan 666303, China; ^3^Hponkan Razi Wildlife Sanctuary Offices, Putao Kachin 01051, Myanmar; ^4^Science Communication and Training Department, Xishuangbanna Tropical Botanical Garden, Chinese Academy of Sciences, Mengla Yunnan 666303, China; ^5^Yingjiang Bird Watching Society, Yingjiang Yunnan 679300, China

**Keywords:** Birds, Hponkan Razi, Hkakabo Razi, Surveys, Conservation, Myanmar

## Abstract

We conducted four bird biodiversity surveys in the Putao area of northern Myanmar from 2015 to 2017. Combined with anecdotal information collected between 2012 and 2015, we recorded 319 bird species, including two species (*Arborophila mandellii* and *Lanius sphenocercus*) previously unrecorded in Myanmar. Bulbuls (Pycnonotidae), babblers (Timaliidae), pigeons and doves (Columbidae), and pheasants and partridges (Phasianidae) were the most abundant groups of birds recorded. Species richness below 1 500 m a. s. l. was higher than species richness at higher elevations. Our results suggest that the current protected areas in this region should be expanded to lower elevations to cover critical conservation gaps.

## INTRODUCTION

The northern part of Myanmar's Kachin State borders both China and India and lies in the Indo-Burma conservation hotspot (Myers et al., 2000). Although this area harbors rich biodiversity, conservation-related research in this region is lacking due to its steep topology, difficult transportation, and variable climate (Rao et al, 2010; Rappole et al., 2011). Since 1990, one new species of mammal (*Muntiacus putaoensis*) (Rabinowitz et al., 1999) and several new subspecies of birds, such as *Tesia olivea chiangmaiensis* (Renner et al., 2008), have been described in this region. This indicates that there are still opportunities for new discovery and exploration and that more information is needed for future conservation plans.

Since the 1990s, several bird surveys had been carried out in the Putao area (Rappole et al, 2011). Under the leadership of the Nature and Wildlife Conservation Division (NWCD) of the Myanmar Forestry Ministry, two expeditions were launched in 1997–1998 (Aung & Oo, 1999) and 2001–2009 (Rappole et al., 2011), providing the most detailed inventory of local avian diversity thus far.

**Figure 1:**
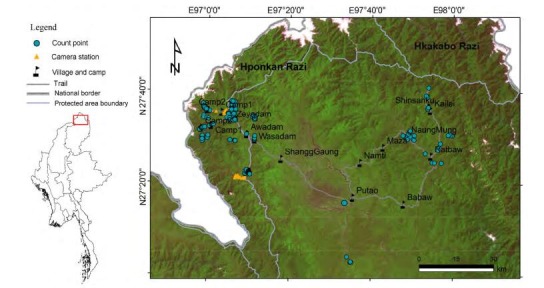
Survey area in Putao, Kachin, northern Myanmar

Between December 2015 and May 2017, the Southeast Asia Biodiversity Research Institute, Chinese Academy of Sciences (CAS-SEABRI), Forest Research Institute (FRI) of Myanmar, Hponkan Razi Wildlife Sanctuary (HPWS), and Hkakabo Razi National Park (HKNP) jointly conducted four general biodiversity surveys in the Putao area of the northwest part of Kachin State, Myanmar. The survey region covered part of HPWS and the surrounding areas south and northeast of Putao (Figure 1). These surveys were conducted to acquire basic biodiversity distribution data for future conservation policy making.

In this paper, we summarize the results of our four surveys as well as episodic observations of a local officer (Myint Kyaw, M.K.). The present research aimed to: (1) update the avian inventory of the study area; and (2) identify survey and conservation gaps in this region.

## MATERIALS AND METHODS

### Study area

The survey area included the southern part of HPWS, one village south of Putao town, and areas northeast of Putao surrounding HKNP (Figure 1). The total survey area was around 5 000 km^2^. Elevation in the area varies from 400 m a.s.l. at Putao to 5 881 m a.s.l. at the Hkakabo Razi Snow Mountain peak (Renner et al., 2007). Vegetation includes lowland tropical rainforest dominated by Dipterocarpaceae between 400 m a.s.l. and 600 m a.s.l., tropical seasonal rainforest dominated by *Terminalia myriocarpa* and *Dysoxylum* sp. from 600 m a.s.l. to 1 200 m a.s.l., mountain tropical rainforest between 1 200 m a.s.l. and 2 000 m a.s.l., subtropical evergreen broad-leaved forest dominated by Lauraceae and Fagaceae between 2 000 m a.s.l. and 2 600 m a.s.l., and bamboo-rhododendron habitats above 3 000 m a.s.l. (Renner et al., 2007; Yun-Hong Tan, personal communication). The temperature varies from 2 ℃ to 36 ℃. Annual precipitation ranges from 3 800–5 100 mm, with a cooler dry season from October to February and a rainy season from March to September (Robinowitz et al., 1999).

### Bird survey

The surveyed habitats include cropland, small areas of riparian wetland (less than 10 count points), and the forest vegetation types mentioned above. Survey locations ranged from 400 m a.s.l. to 3 200 m a.s.l.. We conducted field surveys from 29 November 2015 to 4 January 2016; 23 April to 21 May 2016; 26 November 2016 to 5 January 2017; and 28 April to 26 May 2017.

Point counts and camera traps were combined to obtain bird diversity information. Points were situated along trails from 400 m a.s.l. to 3 200 m a.s.l.; each point was located at least 300 m from other points to ensure quasi-independence (Ralph et al., 1995). We stayed at each point for 12 min, recorded all birds seen or heard within a 100-m radius. We used camera traps to detect ground-dwelling birds and used binoculars to detect all other birds. In total, we established 53 camera stations ranging from 700 m a.s.l. to 3 200 m a.s.l.. The distances between any two camera stations always exceeded 500 m. The cameras were installed in the field between December 2015 and May 2016. The distribution of the point counts and camera traps were mapped out to show the sample effort at different elevations. In addition to systematic data obtained from point counts and camera traps, species observed during field trips were also recorded. The second author M.K., who works at HPWS and HKNP, is an experienced bird-watcher and collected anecdotal information and data through photography and direct observations between 2012 and 2015.

## RESULTS

During the four surveys, we collected information from 304 count points. Of the 53 cameras installed, one was stolen and one was broken. The remaining 51 cameras accumulated more than 7 500 working days of images.

**Figure 2:**
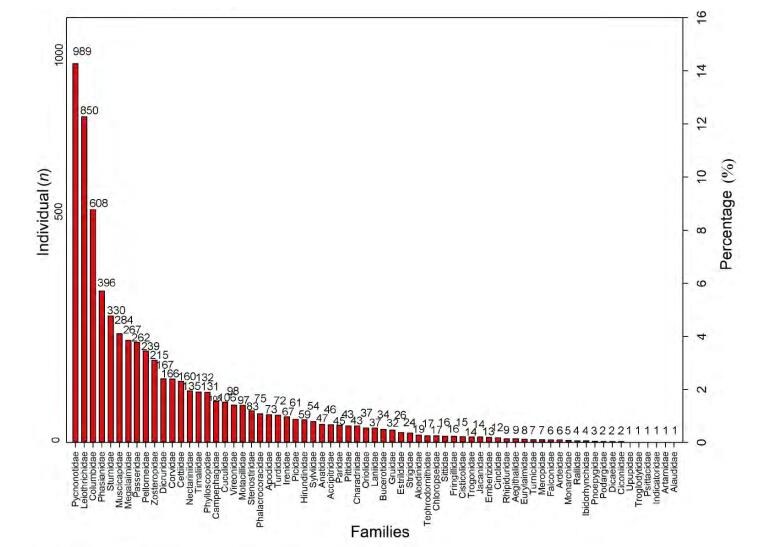
Taxonomic families observed in the bird surveys

**Figure 3:**
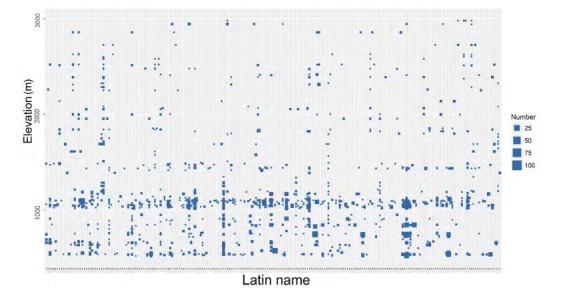
Species distribution in different elevations

**Figure 4:**
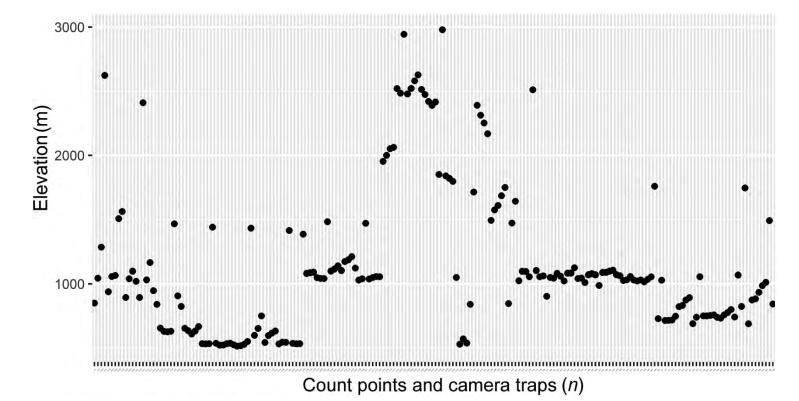
Distribution of count points in different elevations

In total, 319 species were recorded, representing 72 families and 18 orders (Table 1), with one species listed as critically endangered, five species listed as vulnerable, and 13 species listed as near threatened in the IUCN Red List (IUCN, 2016). The percentage of birds of each family is shown in Figure 2. In terms of number of individuals recorded during the surveys, the most abundant families were Pycnonotidae (989 individuals), Leiothrichidae (850 individuals), and Columbidae (608 individuals). Several families recorded, such as Alaudidae, Artamidae, Indicatoridae, Psittacidae, Troglodytidae, and Upupidae, contained only one identified individual. The number of different species at different elevations is shown in Figure 3. The distributions of the point counts and camera traps at different elevations are shown in Figure 4. We observed 3 719 (82%) individuals from 215 (67%) species under 1 500 m a.s.l.. Sampling effort was biased towards elevations below 1 500 m a.s.l..

**Table 1:** Bird species observed in the Putao area of northern Myanmar from 2013 to 2017

Species	English name	IUCN^*^	Rappole et al., 2011^**^	Habitat^***^	Abundance^****^	Source^*****^
ACCIPITRIFORMES						
Accipitridae						
*Accipiter nisus*	Eurasian Sparrowhawk	LC		Fo	+	An
*Accipiter trivirgatus*	Crested Goshawk	LC		Fo, V	+	CP, An
*Accipiter virgatus*	Besra	LC		Fo	+	CP, An
*Aviceda jerdoni*	Jerdon's Baza	LC	×	Fo	+	An
*Aquila heliaca*	Eastern Imperial Eagle	VU	×	Fo	+	An
*Buteo burmanicus*	Himalayan Buzzard	LC		Fi, Fo	+	An
*Buteo japonicus*	Eastern Buzzard	LC	×	Fi	+	An
*Circus cyaneus*	Hen Harrier	LC		Fi	+	An
*Circus melanoleucos*	Pied Harrier	LC		V	+	An
*Gyps himalayensis*	Himalayan Vulture	NT	×	Fo	+	An
*Haliaeetus humilis*	Lesser Fish Eagle	NT	×	Fo, R	+	CP, An
*Ictinaetus malaiensis*	Black Eagle	LC		Fi, Fo	+	An
*Lophotriorchis kienerii*	Rufous-bellied Hawk-Eagle	LC		Fi	+	An
*Milvus migrans*	Black Kite	LC	×	Fi, Fo	+	An
*Nisaetus nipalensis*	Mountain Hawk-Eagle	LC		Fi, Fo	+	CP, An
*Spilornis cheela*	Crested Serpent Eagle	LC		Fi, Fo	+	CP, An
ANSERIFORMES						
Anatidae						
*Mergus merganser*	Common Merganser	LC		R	+	An
*Tadorna ferruginea*	Ruddy Shelduck	LC		R	++	An
APODIFORMES						
Apodidae						
*Cypsiurus balasiensis*	Asian Palm Swift	LC		Fi, V, G	++	CP, An
*Hirundapus caudacutus*	White-throated Needletail	LC	×	V, R	++	CP, An
*Hirundapus giganteus*	Brown-backed Needletail	LC		V, R	++	CP, An
BUCEROTIFORMES						
Bucerotidae						
*Aceros nipalensis*	Rufous-necked Hornbill	VU		Fo	+	CP, An
*Buceros bicornis*	Great Hornbill	NT		Fo	+	An
*Rhyticeros undulatus*	Wreathed Hornbill	LC		Fo	+	CP, An
Upupidae						
*Upupa epops*	Common Hoopoe	LC		V	+	An
CAPRIMULGIFORMES						
Podargidae						
*Batrachostomus hodgsoni*	Hodgson's Frogmouth	LC		Fo	+	An
CHARADRIIFORMES						
Jacanidae						
*Actitis hypoleucos*	Common Sandpiper	LC		Fi, R	+	An
*Calidris temminckii*	Temminck's Stint	LC	×	R	+	An
*Gallinago gallinago*	Common Snipe	LC	×	G	+	CP, An
CHARADRIIFORMES						
Jacanidae						
*Lymnocryptes minimus*	Jack Snipe	LC	×	Fi, R	+	An
*Scolopax rusticola*	Eurasian Woodcock	LC	×	Fo	+	CT
*Tringa ochropus*	Green Sandpiper	LC		R	+	An
*Tringa stagnatilis*	Marsh Sandpiper	LC		R	+	An
Charadriidae						
*Charadrius dubius*	Little Ringed Plover	LC	×	Fi, R	+	An
*Charadrius placidus*	Long-billed Plover	LC	×	Fi, R	+	An
*Vanellus cinereus*	Grey-headed Lapwing	LC	×	G, R, Fi	+	CP, An
*Vanellus duvaucelii*	River Lapwing	NT		G, R, Fi	+	CP, An
*Vanellus indicus*	Red-wattled Lapwing	LC		G, R, Fi	+	CP, An
*Vanellus vanellus*	Northern Lapwing	NT	×	Fi, R	++	CP, An
Ibidorhynchidae						
*Ibidorhyncha struthersii*	Ibisbill	LC		Fi, R	+	An
Turnicidae						
*Turnix suscitator*	Barred Buttonquail	LC	×	Fi	+	CP, An
CICONIIFORMES						
Ciconiidae						
*Ciconia nigra*	Black Stork	LC		Fo, R	+	An
COLUMBIFORMES						
Columbidae						
*Chalcophaps indica*	Emerald Dove	LC	×	Fo	+	CP, An
*Ducula aenea*	Green Imperial Pigeon	LC		Fo	++	CP, An
*Ducula badia*	Mountain Imperial Pigeon	LC		Fo	++++	CP, An
*Macropygia unchall*	Barred Cuckoo-Dove	LC		Fo	+	An
*Spilopelia chinensis*	Spotted Dove	LC		Fi, Fo	++++	CP, An
*Streptopelia orientalis*	Oriental Turtle Dove	LC		Fi, Fo	++++	CP, An
*Streptopelia decaocto*	Eurasian Collared Dove	LC		Fi, Fo	+++	CP, An
*Treron apicauda*	Pin-tailed Green Pigeon	LC		Fo	++++	CP, An
*Treron sphenurus*	Wedge-tailed Green Pigeon	LC		Fo	++	CP, An
CORACIIFORMES						
Alcedinidae						
*Alcedo atthis*	Common Kingfisher	LC		R	+	CP, An
*Alcedo hercules*	Blyth's Kingfisher	NT		R	+	An
*Ceryle rudis*	Pied Kingfisher	LC		R	+	CP
*Halcyon smyrnensis*	White-throated Kingfisher	LC		R	+	CP
*Megaceryle lugubris*	Crested Kingfisher	LC		R	+	CP
Meropidae						
*Merops orientalis*	Green Bee-eater	LC		Fo	+	CP
*Nyctyornis athertoni*	Blue-bearded Bee-eater	LC		Fo	+	CP
CUCULIFORMES						
Cuculidae						
*Cacomantis merulinus*	Plaintive Cuckoo	LC		Fo	+	CP
*Centropus bengalensis*	Lesser Coucal	LC		Fo	+	An
*Clamator coromandus*	Chestnut-winged Cuckoo	LC		Fo	+	CP
*Cuculus canorus*	Common Cuckoo	LC	×	Fo	+	CP
*Cuculus micropterus*	Indian Cuckoo	LC		Fo	++	CP, An
*Cuculus poliocephalus*	Asian Lesser Cuckoo	LC	×	Fo	+	CP, An
*Hierococcyx sparverioides*	Large Hawk-Cuckoo	LC		Fi, Fo	++	CP, An
*Surniculus dicruroides*	Fork-tailed Drongo-Cuckoo	LC		Fo	++	CP, An
FALCONIFORMES						
Falconidae						
*Falco severus*	Oriental Hobby	LC		Fi	+	CP, An
*Falco tinnunculus*	Common Kestrel	LC	×	Fo	+	An
GALLIFORMES						
Phasianidae						
*Arborophila atrogularis*	White-cheeked Partridge	NT		Fo	++	CP, CT, An
*Arborophila mandellii*	Chestnut-breasted Partridge	VU	×	Fo	+	An
*Arborophila rufogularis*	Rufous-throated Partridge	LC		Fo	++++	CP, CT, An
*Arborophila torqueola*	Common Hill Partridge	LC		Fo	++++	CP, CT, An
*Gallus gallus*	Red Junglefowl	LC		G	+	An
*Lophura leucomelanos*	Kalij Pheasant	LC		Fo	++	CT
*Polyplectron bicalcaratum*	Grey Peacock Pheasant	LC		Fo	++	CP, CT, An
*Tragopan blythii*	Blyth's Tragopan	VU		Fo	+	CT
*Tragopan temminckii*	Temminck's Tragopan	LC		Fo	+	CT
GRUIFORMES						
Rallidae						
*Amaurornis phoenicurus*	White-breasted Waterhen	LC	×	G	+	An
Gruidae						
Grus grus	Common Crane	LC		Fi, R	++	CP, An
PELECANIFORMES						
Ardeidae						
*Ardea insignis*	White-bellied Heron	CR		R	+	An
*Ardeola bacchus*	Chinese Pond Heron	LC	×	R	+	An
*Bubulcus Ibis*	Cattle Egret	LC		R	+	An
*Butorides striata*	Striated Heron	LC		R	+	An
*Ixobrychus cinnamomeus*	Cinnamon Bittern	LC	×	R	+	CP
PICIFORMES						
Megalaimidae						
*Megalaima asiatica*	Blue-throated Barbet	LC		Fo	++	CP, An
*Megalaima australis*	Blue-eared Barbet	LC	×	Fo	+	An
*Megalaima franklinii*	Golden-throated Barbet	LC		Fo	+++	CP, An
PICIFORMES						
Megalaimidae						
*Megalaima lineata*	Lineated Barbet	LC		Fo	+	An
*Megalaima virens*	Great Barbet	LC		Fo	++++	CP, An
Picidae						
*Blythipicus pyrrhotis*	Bay Woodpecker	LC		Fo	++	CP, An
*Chrysophlegma flavinucha*	Greater Yellownape	LC		Fo	+	An
*Dendrocopos canicapillus*	Grey-capped Pygmy Woodpecker	LC		Fo	+	An
*Dendrocopos cathpharius*	Crimson-breasted Woodpecker	LC		Fo	+	An
*Dendrocopos darjellensis*	Darjeeling Woodpecker	LC		Fo	+	CT
*Dendrocopos hyperythrus*	Rufous-bellied Woodpecker	LC		Fo	+	An
*Dendrocopos macei*	Fulvous-breasted Woodpecker	LC		Fo	+	CP, An
*Jynx torquilla*	Eurasian Wryneck	LC		V	+	An
*Picus chlorolophus*	Lesser Yellownape	LC		Fo	+	CP, An
*Sasia ochracea*	White-browed Piculet	LC		Fo	+	CP, An
PASSERIFORMES						
Cettiidae						
*Abroscopus albogularis*	Rufous-faced Warbler	LC		Fo	++	CP, An
*Abroscopus schisticeps*	Black-faced Warbler	LC		Fo	+	An
*Abroscopus superciliaris*	Yellow-bellied Warbler	LC	×	Fo	+++	CP, An
*Cettia brunnifrons*	Grey-sided Bush Warbler	LC		Fi	+	An
*Cettia castaneocoronata*	Chestnut-headed Tesia	LC		Fo	++	CP, An
*Horornis fortipes*	Brownish-flanked Bush Warbler	LC	×	G	++	CP, An
*Tesia cyaniventer*	Grey-bellied Tesia	LC		Fo	+	CP
*Tesia olivea*	Slaty-bellied Tesia	LC		Fo	++	CP, An
Sturnidae						
*Acridotheres albocinctus*	Collared Myna	LC		V	++++	An
*Acridotheres tristis*	Common Myna	LC		V	++++	An
*Agropsar sturninus*	Purple-backed Starling	LC	×	V	+	An
*Ampeliceps coronatus*	Golden-crested Myna	LC	×	V	+	An
*Gracula religiosa*	Hill Myna	LC		V	+	An
*Sturnia malabarica*	Chestnut-tailed Starling	LC		G, V	++	CP
Leiothrichidae						
*Actinodura egertoni*	Rusty-fronted Barwing	LC		Fo	++	CP, An
*Actinodura waldeni*	Streak-throated Barwing	LC		Fo	+	CP, An
Aegithalidae						
*Aegithalos bonvaloti*	Black-browed Bushtit	NE	×	Fo	+	CP
*Aegithalos concinnus*	Black-throated Bushtit	LC		Fo	+	CP
Nectariniidae						
*Aethopyga ignicauda*	Fire-tailed Sunbird	LC	×	Fo	+	CP, An
*Aethopyga nipalensis*	Green-tailed Sunbird	LC		Fo	++	CP, An
*Aethopyga saturata*	Black-throated Sunbird	LC		Fo	++	CP, An
PASSERIFORMES						
Nectariniidae						
*Aethopyga siparaja*	Crimson Sunbird	LC		Fo	+	An
*Arachnothera longirostra*	Little Spiderhunter	LC		Fo	+	An
*Arachnothera magna*	Streaked Spiderhunter	LC		Fo	++	CP, An
*Chalcoparia singalensis*	Ruby-cheeked Sunbird	LC	×	Fo	+	An
Alaudidae						
*Alauda gulgula*	Oriental Skylark	LC		G	+	An
Pellorneidae						
*Alcippe castaneceps*	Rufous-winged Fulvetta	LC		Fo	++	CP, An
*Alcippe cinerea*	Yellow-throated Fulvetta	LC		Fo	++	CP, An
*Alcippe morrisonia*	Grey-cheeked Fulvetta	LC		Fo	++	CP, An
*Alcippe nipalensis*	Nepal Fulvetta	LC		Fo	+	CP, An
*Alcippe poioicephala*	Brown-cheeked Fulvetta	LC		Fo	+	CP, An
*Alcippe rufogularis*	Rufous-throated Fulvetta	LC		Fo	+	CP, An
Motacillidae						
*Anthus hodgsoni*	Olive-backed Pipit	LC		Fi, Fo	++	CP, An
*Anthus richardi*	Richard's Pipit	LC		V	+	An
*Anthus roseatus*	Rosy Pipit	LC	×	Fi	++	An
*Anthus rufulus*	Paddyfield Pipit	LC	×	Fi	+	An
*Motacilla alba*	White Wagtail	LC		R	++	CP, An
*Motacilla cinerea*	Grey Wagtail	LC		V	+	An
*Motacilla citreola*	Citrine Wagtail	LC		V	+	An
Artamidae						
*Artamus fuscus*	Ashy Woodswallow	LC		V	+	An
Elachuridae						
*Elachura formosa*	Spotted Wren-babbler	LC	×	Fo	+	An
Muscicapidae						
*Anthipes monileger*	White-gorgeted Flycatcher	LC		Fo	+	An
*Brachypteryx hyperythra*	Rusty-bellied Shortwing	NT		Fo	+	CP
*Brachypteryx montana*	White-browed Shortwing	LC		Fo	+	An, CT
*Chaimarrornis leucocephalus*	White-capped Water Redstart	LC		R	++	CP, An
*Copsychus saularis*	Oriental Magpie Robin	LC		V, Fi, G	++	CP, An
*Cyornis banyumas*	Hill Blue Flycatcher	LC		Fo	++	CP, An
*Cyornis rubeculoides*	Blue-throated Flycatcher	LC		Fo	+	An
*Cyornis tickelliae*	Tickell's Thrush	LC		Fo	+	An
*Enicurus leschenaulti*	White-crowned Forktail	LC		R	+	An
*Enicurus maculatus*	Spotted Forktail	LC		R	+	CP, An
*Enicurus schistaceus*	Slaty-backed Forktail	LC		R	++	CP, An
*Enicurus scouleri*	Little Forktail	LC		R	+	CP, An
*Eumyias thalassinus*	Verditer Flycatcher	LC		Fo	+	CP
*Ficedula albicilla*	Taiga Flycatcher	LC	×	Fo	+	CP, An
PASSERIFORMES						
Muscicapidae						
*Ficedula hyperythra*	Snowy-browed Flycatcher	LC		Fo	+	An
*Ficedula strophiata*	Rufous-gorgeted Flycatcher	LC		Fo	+	CP, An
*Ficedula superciliaris*	Ultramarine Flycatcher	LC	×	Fo	+	CP
*Luscinia svecica*	Bluethroat	LC	×	Fo	+	An
*Monticola solitarius*	Blue Rock Thrush	LC		R	+	CP
*Muscicapa ferruginea*	Ferruginous Flycatcher	LC	×	Fo	+	CP
*Myiomela leucura*	White-tailed Robin	LC		Fo	+	CT
*Myophonus caeruleus*	Blue Whistling Thrush	LC		Fo, V, R	+++	CP, An
*Niltava grandis*	Large Niltava	LC		Fo	++	CP, An
*Niltava macgrigoriae*	Small Niltava	LC		Fo	+	CP, An
*Niltava sundara*	Rufous-bellied Niltava	LC		Fo	+	CP, An
*Phoenicurus auroreus*	Daurian Redstart	LC		Fo, V	+	CP
*Phoenicurus hodgsoni*	Hodgson's Redstart	LC	×	V	+	CP, An
*Rhyacornis fuliginosa*	Plumbeous Water Redstart	LC		R	++	CP, An
*Saxicola caprata*	Pied Bushchat	LC		Fo	+	An
*Saxicola ferreus*	Grey Bushchat	LC		Fo	+	CP, An
*Saxicola torquatus*	Siberian Stonechat	LC		V	+	An
*Tarsiger hyperythrus*	Rufous-breasted Bush Robin	LC	×	Fo	+	An
*Tarsiger indicus*	White-browed Bush Robin	LC	×	Fo	++	CT
Campephagidae						
*Coracina melaschistos*	Black-winged Cuckooshrike	LC		Fo	++	CP, An
*Pericrocotus brevirostris*	Short-billed Minivet	LC		Fo	++	CP, An
*Pericrocotus ethologus*	Long-tailed Minivet	LC		Fo	+	CP
*Pericrocotus roseus*	Rosy Minivet	LC		Fo	+	CP
*Pericrocotus solaris*	Grey-chinned Minivet	LC		Fo	++	CP, An
*Pericrocotus speciosus*	Scarlet Minivet	LC		Fo	+	CP
Chloropseidae						
*Chloropsis hardwickii*	Orange-bellied Leafbird	LC		Fo	++	CP, An
Cinclidae						
*Cinclus pallasii*	Brown Dipper	LC		R	++	CP, An
Cisticolidae						
*Cisticola juncidis*	Zitting Cisticola	LC	×	V	+	An
*Orthotomus sutorius*	Common Tailorbird	LC		G, V	+	CP, An
*Prinia crinigera*	Striated Prinia	LC	×	Fo	+	CP
*Prinia inornata*	Plain Prinia	LC	×	G	+	An
*Prinia rufescens*	Rufescent Prinia	LC	×	G	+	An
*Prinia superciliaris*	Hill Prinia	LC		G	+	An
Corvidae						
*Cissa chinensis*	Common Green Magpie	LC		Fo	++	CP
*Corvus macrorhynchos*	Large-billed Crow	LC		Fo, V	+++	CP, An
PASSERIFORMES						
Corvidae						
*Dendrocitta formosae*	Grey Treepie	LC		Fi, Fo	+++	CP, An
*Dendrocitta frontalis*	Collared Treepie	LC		Fo, V	++	CP, An
*Garrulus glandarius*	Eurasian Jay	LC		Fo	+	An
*Urocissa flavirostris*	Yellow-billed Blue Magpie	LC		Fo	+	An
Dicaeidae						
*Dicaeum minullum*	Plain Flowerpecker	LC		Fo	+	CP
Dicruridae						
*Dicrurus aeneus*	Bronzed Drongo	LC		Fo	++	CP, An
*Dicrurus hottentottus*	Hair-crested Drongo	LC		Fo	++	CP, An
*Dicrurus leucophaeus*	Ashy Drongo	LC		Fo	++	CP, An
*Dicrurus macrocercus*	Black Drongo	LC		Fi, Fo	++++	CP, An
*Dicrurus paradiseus*	Greater Racket-tailed Drongo	LC		Fo	+	An
*Dicrurus remifer*	Lesser Racket-tailed Drongo	LC		Fi, Fo	+	CP, An
Emberizidae						
*Emberiza pusilla*	Little Bunting	LC		Fi	++	CP, An
Estrildidae						
*Lonchura striata*	White-rumped Munia	LC		Fi, G	++	CP, An
Eurylaimidae						
*Psarisomus dalhousiae*	Long-tailed Broadbill	LC		Fo, V	+	An
*Serilophus lunatus*	Silver-breasted Broadbill	LC		Fo	+	An
Fringillidae						
*Carpodacus erythrinus*	Common Rosefinch	LC		G	+	CP, An
*Procarduelis nipalensis*	Dark-breasted Rosefinch	LC		G	++	CP, An
Hirundinidae						
*Hirundo rustica*	Barn Swallow	LC		V	+	An
*Riparia chinensis*	Asian Plain Martin	NE		R	+++	CP, An
Indicatoridae						
*Indicator xanthonotus*	Yellow-rumped Honeyguide	NT	×	Fo	+	An
Irenidae						
*Irena puella*	Asian Fairy Bluebird	LC		V	+++	An
Laniidae						
*Lanius collurioides*	Burmese Shrike	LC		V	+	An
*Lanius cristatus*	Brown Shrike	LC		V	+	An
*Lanius schach*	Long-tailed Shrike	LC		Fo, V	++	CP, An
*Lanius sphenocercus*	Chinese Grey Shrike	LC	×	V	+	An
*Lanius tephronotus*	Grey-backed Shrike	LC		Fo, V	+	CP, An
Leiothrichidae						
*Cutia nipalensis*	Himalayan Cutia	LC		Fo	+	CP, An
*Garrulax caerulatus*	Grey-sided Laughingthrush	LC	×	Fo	++	CP, CT
*Garrulax leucolophus*	White-crested Laughingthrush	LC		Fo	++++	CP, CT, An
PASSERIFORMES						
Leiothrichidae						
*Garrulax monileger*	Lesser Necklaced Laughingthrush	LC		Fo	+	CP
*Garrulax pectoralis*	Greater Necklaced Laughingthrush	LC		Fo	++	CP, CT
*Garrulax ruficollis*	Rufous-necked Laughingthrush	LC	×	Fo	++	CP, An
*Garrulax rufogularis*	Rufous-chinned Laughingthrush	LC		Fo	+	CP
*Garrulax striatus*	Striated Laughingthrush	LC		Fo	++	CP, An
*Garrulax nuchalis*	Chestnut-backed Laughingthrush	NT		Fo	+	An
*Heterophasia annectans*	Rufous-backed Sibia	LC		Fo	+	CP, An
*Heterophasia picaoides*	Long-tailed Sibia	LC		Fo	++	CP, An
*Heterophasia pulchella*	Beautiful Sibia	LC		Fo	++	CP, An
*Leiothrix argentauris*	Silver-eared Mesia	LC		Fo, G	++++	CP, An
*Leiothrix lutea*	Red-billed Leiothrix	LC		Fo	+	An
*Minla cyanouroptera*	Blue-winged Minla	LC		Fo	+++	CP, An
*Minla ignotincta*	Red-tailed Minla	LC		Fo	++	CP, An
*Minla strigula*	Bar-throated Minla	LC		Fo	++	CP, An
*Trochalopteron affine*	Black-faced Laughingthrush	LC		Fo	++	CT
*Trochalopteron squamatum*	Blue-winged Laughingthrush	LC		Fo	++	CT
*Trochalopteron subunicolor*	Scaly Laughingthrush	LC		Fo	++	CT
*Liocichla phoenicea*	Red-faced Liocichla	LC		Fo	+	An
Locustellidae						
*Megalurus palustris*	Striated Grassbird	LC		G	+	An
Monarchidae						
*Hypothymis azurea*	Black-naped Monarch	LC		Fo	+	CP
*Terpsiphone paradisi*	Indian Paradise Flycatcher⑧	LC		Fo	+	CP, An
Oriolidae						
*Oriolus traillii*	Maroon Oriole	LC		Fo	++	CP, An
Paridae						
*Machlolophus spilonotus*	Yellow-cheeked Tit	LC		Fo	+	CP, An
*Melanochlora sultanea*	Sultan Tit	LC		Fo	+	An
*Parus monticolus*	Green-backed Tit	LC		Fo	++	CP, An
*Periparus ater*	Coal Tit	LC		Fo	+	An
*Periparus rubidiventris*	Rufous-vented Tit	LC		Fo	+	CP
Passeridae						
*Passer domesticus*	House Sparrow	LC		V	++	An
*Passer montanus*	Eurasian Tree Sparrow	LC		G, V	++++	CP, An
*Passer rutilans*	Russet Sparrow	LC	×	V	+	An
Pellorneidae						
*Gampsorhynchus rufulus*	White-hooded Babbler	LC		Fo	+	CP
*Pellorneum albiventre*	Spot-throated Babbler	LC		Fo	+	CP
*Pellorneum ruficeps*	Puff-throated Babbler	LC		Fo	+	CP
PASSERIFORMES						
*Phylloscopidae*						
*Phylloscopus cantator*	Yellow-vented Warbler	LC		Fo	+	CP
*Phylloscopus claudiae*	Claudia's Leaf Warbler	LC	×	Fo	++	CP, An
*Phylloscopus davisoni*	Davison's Leaf Warbler	LC	×	Fo	+	An
*Phylloscopus fuscatus*	Dusky Warbler	LC		Fo	+	An
*Phylloscopus maculipennis*	Ashy-throated Warbler	LC		Fo	+	CP
*Phylloscopus reguloides*	Blyth's Leaf Warbler	LC		Fo	+	CP
*Phylloscopus whistleri*	Whistler's Warbler	LC	×	Fo	+	An
*Seicercus affinis*	White-spectacled Warbler	LC		Fo	++	CP, An
*Seicercus burkii*	Green-crowned Warbler	LC		Fo	++	CP, An
*Seicercus castaniceps*	Chestnut-crowned Warbler	LC		Fo	++	CP, An
*Seicercus poliogenys*	Grey-cheeked Warbler	LC		Fo	++	CP, An
*Seicercus tephrocephalus*	Grey-crowned Warbler	LC		Fo	+	An
Pittidae						
*Hydrornis nipalensis*	Blue-naped Pitta	LC	×	Fo	++	CT
Pnoepygidae						
*Pnoepyga pusilla*	Pygmy Wren-Babbler	LC		Fo	+	CP, An
Pycnonotidae						
*Alophoixus flaveolus*	White-throated Bulbul	LC		Fo	+++	CP, An
*Alophoixus pallidus*	Puff-throated Bulbul	LC	×	Fo	++	CP, An
*Hemixos flavala*	Ashy Bulbul	LC		Fo	++	CP, An
*Hypsipetes leucocephalus*	Black Bulbul	LC		Fo, V	++++	CP, An
*Iole propinqua*	Grey-eyed Bulbul	LC	×	Fo	+	An
*Ixos mcclellandii*	Mountain Bulbul	LC		Fo	+	CP
*Pycnonotus cafer*	Red-vented Bulbul	LC		Fo, V, Fi, G	+++	CP, An
*Pycnonotus flaviventris*	Black-crested Bulbul	LC		Fo	++	CP, An
*Pycnonotus jocosus*	Red-whiskered Bulbul	LC		Fo, V, Fi, G	++++	CP, An
*Pycnonotus striatus*	Striated Bulbul	LC		G	+	An
Rhipiduridae						
*Rhipidura albicollis*	White-throated Fantail	LC		Fo	+	CP, An
Sittidae						
*Sitta cinnamoventris*	Chestnut-bellied Nuthatch	LC		Fo	++	CP, An
*Sitta frontalis*	Velvet-fronted Nuthatch	LC		Fo	+	CP, An
*Sitta himalayensis*	White-tailed Nuthatch	LC		Fo	+	An
Stenostiridae						
*Chelidorhynx hypoxantha*	Yellow-bellied Fantail	LC		Fo	++	CP, An
*Culicicapa ceylonensis*	Grey-headed Canary Flycatcher	LC		Fo, V	++	CP, An
Strigidae						
*Glaucidium brodiei*	Collared Owlet	LC		Fo	+	An
*Glaucidium cuculoides*	Asian Barred Owlet	LC		Fo, V	++	CP, An
*Otus spilocephalus*	Mountain Scops Owl	LC		Fo, V	+	An
PASSERIFORMES						
Sylviidae						
*Chleuasicus atrosuperciliaris*	Lesser Rufous-headed Parrotbill	LC		Fo	++	CP, An
*Lioparus chrysotis*	Golden-breasted Fulvetta	LC	×	Fo	+	CP
*Psittiparus bakeri*	Rufous-headed Parrotbill	LC		Fo	+	CP, An
*Psittiparus gularis*	Grey-headed Parrotbill	LC		Fo	+	CP, An
*Suthora poliotis*	Grey-breasted Parrotbill	LC		Fo	++	CP, An
Tephrodornithidae						
*Hemipus picatus*	Bar-winged Flycatcher-shrike	LC		Fo	++	CP
Timaliidae						
*Pomatorhinus ferruginosus*	Coral-billed Scimitar Babbler	LC		Fo	++	CP, An
*Pomatorhinus ochraceiceps*	Red-billed Scimitar Babbler	LC		Fo	+	CP
*Pomatorhinus ruficollis*	Streak-breasted Scimitar Babbler	LC	×	Fo	+	CP
*Stachyris roberti*	Chevron-breasted Babbler	N/A		Fo	++	CT
*Stachyridopsis chrysaea*	Golden Babbler	LC		Fo	++	CP, An
*Stachyridopsis ruficeps*	Rufous-capped Babbler	LC		Fo	++	CP
*Stachyridopsis rufifrons*	Rufous-fronted Babbler	LC		Fo	+	An
*Stachyris nigriceps*	Grey-throated Babbler	LC		Fo	++	CP, An
*Stachyris oglei*	Snowy-throated Babbler	VU		Fo	+	An
Troglodytidae						
*Troglodytes troglodytes*	Eurasian Wren	LC	×	Fo	+	An
*Harpactes erythrocephalus*	Red-headed Trogon	LC		Fo	+	CT
*Harpactes wardi*	Ward's Trogon	NT	×	Fo	+	An
Turdidae						
*Cochoa viridis*	Green Cochoa	LC	×	Fo	+	An
*Turdus boulboul*	Grey-winged Blackbird	LC		Fo	+	An
*Zoothera mollissima*	Plain-backed Thrush	LC	×	Fo	+++	An, CT
Vireonidae						
*Erpornis zantholeuca*	White-bellied Erpornis	LC		Fo	++	CP, An
*Pteruthius aeralatus*	White-browed Shrike Babbler	LC		Fo	+	CP
*Pteruthius aenobarbus*	Chestnut-fronted Shrike Babbler	LC	×	Fo		An
*Pteruthius melanotis*	Black-eared Shrike Babbler	LC		Fo	++	CP, An
*Pteruthius xanthochlorus*	Green Shrike Babbler	LC		Fo	+	CP
Zosteropidae						
*Yuhina bakeri*	White-naped Yuhina	LC		Fo	+++	CP, An
*Yuhina castaniceps*	Striated Yuhina	LC		Fo	++	CP, An
*Yuhina flavicollis*	Whiskered Yuhina	LC		Fo	++	CP, An
*Yuhina gularis*	Stripe-throated Yuhina	LC		Fo	++	CP, An
*Yuhina nigrimenta*	Black-chinned Yuhina	LC		Fo	++	CP, An
*Zosterops erythropleurus*	Chestnut-flanked White-eye	LC	×	Fo	+	CP, An
*Zosterops palpebrosus*	Oriental White-eye	LC		Fo	+	CP, An
PSITTACIFORMES						
Psittacidae						
*Psittacula finschii*	Grey-headed Parakeet	NT		V	+	An
SULIFORMES						
Phalacrocoracidae						
*Phalacrocorax carbo*	Great Cormorant	LC		R	+++	CP, An
^*^ LC: Least Concern; NT: Near Threatened; VU: Vulnerable; CR: Critically Endangered; NE: Not Evaluated.^**^ "×" means the species was recorded in this study, but not in Rappole et al. (2011). ^***^ Fo: Forest; V: Village; Fi: Field; R: River; G: Grassland. ^****^ "+" means the number is between 1–10; "++" means the number is between 11–50; "+++" means the number is between 51–100; "++++" means the number is more than 100. ^*****^ An: Anecdotal observation; CP: Counting point; CT: Camera trap.						

### New record

We observed three new bird records for northern Myanmar (*Agropsar sturninus*, *Aviceda jerdoni*, and *Ampeliceps coronatus*) and two new record species (*Arborophila mandellii* and *Lanius sphenocercus*) for Myanmar.

### Purple-backed Starling (*Agropsar sturninus*)

**Figure 5:**
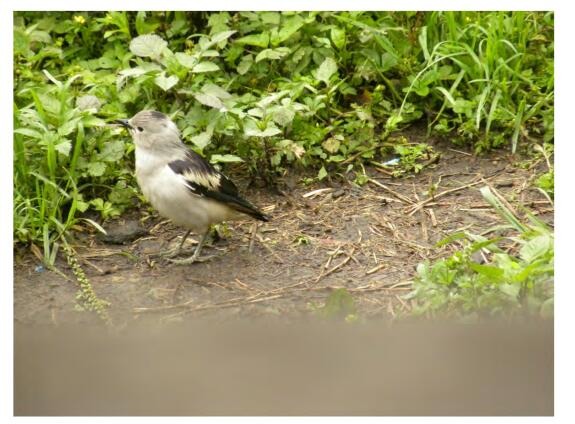
Photograph of Purple-backed Starling (*Agropsar sturninus*) (photo by Myint Kyaw)

One individual was photographed (Figure 5) by M.K. on 30 April 2012 in Makung Ghang Station (E98°16′56″, N27°38′54″) in the Hkakabo Razi direction.

### Jerdon's Baza (*Aviceda jerdoni*)

One individual was photographed by Z.X.L. (fifth author) on 5 May 2016 around camp 2 at Hponyin Razi (E96°59′3″, N27°36′10″).

### Golden-crested Myna (*Ampeliceps coronatus*)

One individual was photographed by M.K. on 23 November 2013 at Ziadam village (E97°5′56″, N27°34′13″).

### Chestnut-breasted Partridge (*Arborophila mandellii*)

**Figure 6:**
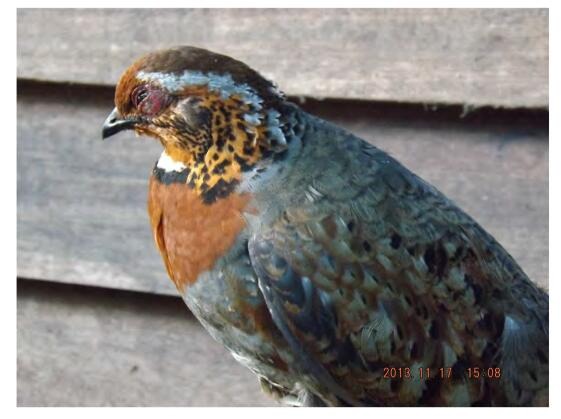
Photograph of Chestnut-breasted Partridge (*Arborophila mandellii*) (photo by Myint Kyaw)

One individual was photographed (Figure 6) by M.K. on 17 November 2013. The bird was hunted by a local villager close to camp 1 at Hponyin Razi (E96°58′52″, N27°36′21″).

### Chinese Grey Shrike (*Lanius sphenocercus*)

**Figure 7:**
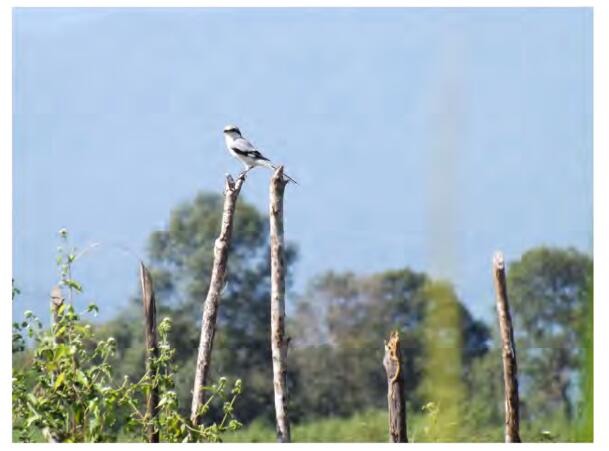
Photograph of Chinese Grey Shrike (*Lanius sphenocercus*) (photo by Myint Kyaw)

One individual was photographed (Figure 7) by M.K. on 23 November 2013 at Jobali village close to Putao town (E97°35′, N27°2′). It was identified by its large body size, black facial mask, grey nape and upperparts, white scapular, and white spot on the wings.

## DISCUSSION

Because our data came from direct observations and camera trap photos, some species could be under sampled, particularly those living in shrub and canopy habitats. Our sampling effort was more intensive in lower elevations, partly because a larger proportion of the survey area occurred at lower elevations and partly because of easier transportation in the lowland plain. The overrepresentation of lower elevation habitats in our study could explain the low frequency of many bird families in our surveys. In the future, surveys should be conducted at higher elevations, especially in the mountains above 3 000 m a.s.l.. Although our sampling effort and checklist is incomplete, the present work provides preliminary data for local diversity research and conservation projects.

Rappole et al. (2011) conducted five bird surveys in a 10 000 km^2^ area in the Hkakabo Razi region, mainly covering the east. While the present study area also lies within the same region, we focused on the western part of Putao. Furthermore, Rappole et al. (2011) set mist nets from 500–3 000 m a.s.l., and recorded 413 species, of which 159 were not observed during our surveys. Of the 319 species recorded in the present research, 65 were not recorded by Rappole et al. (2011). The greatest difference in species occurred in the families Muscicapidae (*n*=7) and Accipitridae (*n*=6). This disparity between studies is likely explained by the different study areas sampled as well as the differences in methodology. Our methodology was more suited for detecting certain species. For example, birds of prey (Accipitridae) can be more easily observed during point counts, and some cryptic species can be captured by camera trap. In our study, one Eurasian Woodcock (*Scolopax rusticola*) was captured by camera trap at 1 190 m a.s.l.. We believe future exploration of new survey routes and a combination of different survey methods will lead to the discovery of additional species not previously recorded here.

The Chestnut-breasted Partridge was identified by its rufescent crown, white gorget, and chestnut breast. It was previously only known from Bhutan, Southeast Tibet, China, and Northeast India (McGowan et al., 2017). The Chinese Grey Shrike was previously recorded in eastern Mongolia to southeast Russia, northern China, and North and South Korean, also known to migrate to east and southeast China and Korea (Yosef & International Shrike Working Group, 2017). The finding of these species formerly unrecorded in the area not only increase two new species of birds for Myanmar, but also reiterates the high biodiversity in this region and highlights the importance of long-term field observations.

Based on our observations, the forest is in good condition. The Leiothrichidae, Columbidae, and Phasianidae species are forest-adapted, and their high abundance shows adequate food and resources for breeding in the local forest. Hornbills were observed almost every day around the camp sites at 1000–1 500 m a.s.l. during winter, and gibbons (*Hoolock* spp.) were often heard from the camp sites below 2 000 m a.s.l.. The most abundant species observed belonged to Pycnonotidae, Leiothrichidae, Columbidae, and Phasianidae. The bulbuls (Pycnonotidae) are common birds living in tropical and subtropical areas, which can adapt to diverse habitats (Fishpool & Tobias, 2017).

In addition to the forest, the local cropland also provides important habitat for many migrating species. Farmers in Putao plant large areas of rice in June-July and harvest it in October-November. The land is barren in other months of the year. These fallow lands provide important habitat for migrating bird species. M.K. observed more than 10 000 common cranes (*Grus grus*) migrating through Putao in March 2016, where they forage in the fallow rice paddies and wetlands by the Malika riverside.

Figure 3 shows that both abundance and species richness were higher below 1 500 m a.s.l.. Species of high conservation value, (e.g., critically-endangered White-bellied Heron (*Ardea insignis*), vulnerable Rufous-necked Hornbill (*Aceros nipalensis*), and near-threatened Lesser Fish Eagle (*Haliaeetus humilis*)) were all recorded under 1 500 m a.s.l.. We also observed some birds with narrow ranges such as the Snowy-throated Babbler (*Stachyris oglei*) at lower elevations. Current protected area boundaries are set too high in minimal elevation (1 000 m a.s.l. for HPWS and 900 m a.s.l. for HKNP) and should be lowered to accommodate greater biodiversity, a conclusion also drawn by former researchers (Rabinowitz et al., 1999; Renner et al., 2007). The Burmese government currently plans to extend these two protected areas to incorporate lower elevations, and is applying for World Heritage Site status for these two regions. This is an important decision with long-term benefits for local conservation. The HPWS forest connects to the Hukawng Valley extension in the west. Extending the current HPWS and HKNP borders will ensure the protection of the largest forest complex in northern Myanmar and will maintain intact habitat for important wildlife.

## ACKNOWLEDGEMENTS

We are grateful for the support from the HPWS and HKNP Offices and FRI of Myanmar during field work. We thank Mr. Kyaw Win Maung, Mr. Kyaw Win Myint, and Mr. Dee Shin for their help during field work. We appreciate Mr. Francis Commercon for improving the language of the manuscript.
